# Entrainment during Ablation of Ischemic Ventricular Tachycardia. What is explanation for Post Pacing Interval shorter than the tachycardia cycle length?

**Published:** 2011-11-15

**Authors:** Kartikeya Bhargava, Hariraj Singh Tomar, Pankaj Gupta, Balbir Singh

**Affiliations:** Division of Cardiac Electrophysiology and Pacing, Medanta Heart Institute, Medanta, The Medicity, Gurgaon, Delhi NCR, India

**Keywords:** entrainment, post-pacing interval, ventricular tachycardia

## Abstract

Entrainment mapping of ischemic ventricular tachycardia at a site in the left ventricle where radiofrequency ablation was successful in terminating the tachycardia revealed a post-pacing interval shorter than the tachycardia cycle length. The reason for the same is explained in the current report.

## Case Report

**Figure 1:**
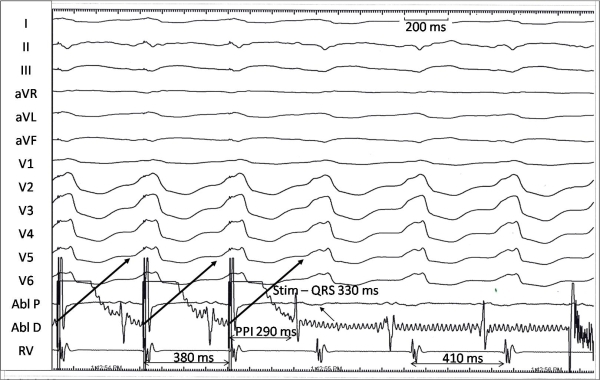
Twelve lead ECG along with ablation proximal (Abl P), ablation distal (Abl D) and right ventricular apical (RV) intracardiac electrograms during attempted entrainment of ischemic ventricular tachycardia. The ablation signals were sub-optimal in the proximal channel due to technical reasons and the distal channel shows noise due to electrical interference during ablation. PPI is post pacing interval. Stim-QRS is stimulus to QRS interval.

A 65-year old gentleman, a known case of coronary artery disease and severe left ventricular dysfunction underwent radiofrequency catheter ablation for recurrent shocks from implantable defibrillator due to frequent ventricular tachycardia (VT). The consent for the procedure and for the publication was taken from the patient. The procedure was done using CARTO XP mapping system through retrograde aortic approach. A monomorphic ventricular tachycardia with cycle length 410 ms, right bundle branch block morphology, superior axis of QRS in the frontal plane and positive concordance of QRS in the precordial leads was easily and repeatedly inducible. An activation map of the VT was created in the left ventricle and entrainment was attempted from an area on the anterolateral wall near the mitral annulus. Pacing was performed from the ablation catheter at 380 ms during VT (Figure 1). There was entrainment with concealed fusion with an identical QRS morphology during pacing with an apparent post pacing interval (PPI) of 290 ms and stimulus to QRS of 330 ms. How can one explain a PPI shorter than the tachycardia cycle length during entrainment with concealed fusion? Radiofrequency ablation at this site resulted in termination of the tachycardia.

A careful look at the figure reveals that the cycle length of the tachycardia shortens to 380 ms during attempted entrainment with an identical QRS morphology. However, the electrogram in the ablation catheter that is used to measure PPI is present earlier than the pacing stimulus and is not altered during pacing implying that it is a far field signal and not true near field electrogram.[1] The large amplitude of this electrogram, possibly due to neighboring anterolateral papillary muscle, in no way excludes the possibility of it being a far field signal. The near field electrogram cannot be made out in the ablation catheter due to artifacts (due to enabling of RF energy) after pacing was stopped. It was not discernible at this site both in the distal and proximal bipole even when the pacing was disabled (not shown) probably because of very small amplitude of the true local electrogram. Though, there is entrainment with concealed fusion, absence of a discernible local electrogram in the ablation channel precludes measurement of true PPI. Thus, this site could be in the reentry circuit. Though, a long stimulus to QRS interval of 330 ms (80% of the tachycardia cycle length) points to an inner loop site, [2]  it can also be in the critical isthmus or the exit site with very slow conduction. Though, the chances of termination of VT at a site with stimulus to QRS interval greater than 70% of VT cycle length are small, ablation at this site successfully terminated the VT in this case. Scar related VT usually needs a linear ablation for termination, however, a single lesion delivered in the narrow critical isthmus may terminate the tachycardia as in the present case. The ablation line was created joining the successful site of ablation to the mitral annulus. The present case suggests that it is important to identify far field signals during ablation of ischemic VT and entrainment can help in their recognition.
